# Severe refractory hypotension during induction of general anesthesia in patient after 48 hours of azilsartan discontinuation: A case report

**DOI:** 10.1097/MD.0000000000036126

**Published:** 2023-11-24

**Authors:** Ji Hye Lee, Yu Yil Kim, Hyun Joo Heo, Gwanbeom Kim, Changhwan Oh

**Affiliations:** a Department of Anesthesiology and Pain Medicine, Presbyterian Medical Center, Jeonju, Jeollabuk-Do, Korea.

**Keywords:** angiotensin receptor blocker, azilsartan, case report, general anesthesia, hypotension, refractory

## Abstract

**Rationale:**

Angiotensin II receptor blockers (ARBs) are currently considered first-line antihypertensive drugs, effectively inhibiting the renin-angiotensin-aldosterone system. However, ARBs have been associated with intraoperative hypotension during general anesthesia. Although it is recommended to discontinue ARBs for 24 hours before surgery, the optimal duration of discontinuation remains unclear. We present a severe refractory hypotension encountered during general anesthesia despite discontinuing ARBs for 48 hours before anesthesia.

**Patient concerns:**

A severe refractory hypotension occurred during the induction of general anesthesia for cranioplasty in a 66-year-old male patient (170 cm/75 kg). The patient was taking azilsartan, angiotensin receptor blocker, for hypertension, which was discontinued 48 hours before anesthesia induction. Despite repeated administration of ephedrine and continuous infusion of norepinephrine, hemodynamic instability did not improve. Therefore, the surgery was postponed.

**Diagnosis:**

The patient was diagnosed with angiotensin receptor blocker-induced refractory hypotension.

**Interventions:**

Before the second surgery, the angiotensin receptor blocker was discontinued 96 hours prior to the surgery. Invasive blood pressure monitoring was performed before anesthesia induction, and vasopressin was prepared. General anesthesia was induced using remimazolam and maintained with desflurane.

**Outcomes:**

The surgery was completed successfully without occurrence of refractory hypotension.

**Lessons:**

Refractory hypotension induced by Angiotensin receptor blockers can still occur even after discontinuing the medication for 48 hours before induction of general anesthesia. Despite withholding the medication, caution should be practiced regarding hypotension during general anesthesia in patient taking ARBs.

## 1. Introduction

Angiotensin II receptor blockers (ARBs) that inhibit the renin-angiotensin-aldosterone system (RAAS) are currently considered first-line hypertensive drugs.^[1]^ ARBs have been associated with intraoperative hypotension during general anesthesia.^[2]^ Although, there is no clear consensus on whether to discontinue or continue these medications prior to general anesthesia in patients chronically using ARBs, it is generally recommended to discontinue ARBs for 24 hours before anesthesia induction.^[1,3–5]^ However, despite discontinuing ARBs for 24 hours before surgery, there have been consistent reports of refractory hypotension.^[6–9]^ Furthermore, there have been reports that prolonging the discontinuation period of ARBs to 2 to 5 days resulted in successful hemodynamic stability during general anesthesia.^[7–9]^ Herein, we present a case report of severe refractory hypotension encountered during general anesthesia induction after a 48-hour discontinuation of azilsartan.

## 2. Case presentation

The patients’ medical records were reviewed following ethical approval, and the requirement for informed consent was waived by the Institutional Review Board of the Presbyterian Medical Center (IRB no. E2023-02).

A 66-year-old male patient (170 cm/75 kg) was scheduled to undergo a cranioplasty under general anesthesia. The patient underwent 2 emergency craniectomy for subdural hemorrhage 2 months ago. The patient had well-controlled hypertension and diabetes. Antihypertensive medications included azilsartan, amlodipine, and carvedilol. During the pre-anesthetic evaluation, the blood pressure (BP) in general ward was measured 110/70 mm Hg, the heart rate (HR) was 68 bpm, and the oxygen saturation was 98%. Chest x-ray was normal. Electrocardiogram showed a normal sinus rhythm with a HR of 61 bpm. Echocardiogram revealed an ejection fraction of 66%, and no abnormalities were observed in the heart wall motions and valves. The laboratory test results were within normal range. Azilsartan, an angiotensin receptor blocker, was discontinued the day before surgery.

Standard monitoring devices were applied upon arrival in the operating room, including noninvasive blood pressure, electrocardiogram, pulse oximetry, and patient state index. The initial vital signs were stable, with a BP of 139/87 mm Hg, HR of 68 bpm, and oxygen saturation of 95% at room air. After pre-oxygenation, anesthesia induction was performed using propofol 130 mg, remifentanil (an effect-site concentration of 1.0 ng/mL) via target-controlled infusion, rocuronium 60 mg. Desflurane at 6 vol% concentration and oxygen at 6 L/min were administered for mask ventilation. Severe hypotension occurred before endotracheal intubation with BP of 64/31 mm Hg and HR of 64 bpm. Endotracheal intubation was performed immediately. However, BP further decreased to 49/21 mm Hg (HR 63 bpm). Despite administering ephedrine 10 mg twice and loading colloid fluid, BP remained low at 40-50/20-30 mm Hg. Epinephrine was intravenously administered at a dose 100 mcg twice, and BP increased to 103/57 mm Hg. However, it subsequently decreased to 70/42 mm Hg. BP was maintained 60-80/40-50 mm Hg with the continuous infusion of norepinephrine. During this time, HR ranged 63 to 70 bpm. The decision to postpone the surgery was made, and after emergence from anesthesia, the patient was transferred to the recovery room. After approximately 2 hours, BP recovered and stabilized. An assessment of the severe hypotension during induction of anesthesia was conducted. Electrocardiogram and echocardiogram were no significant changes. Cardiac enzymes were within normal range. The anesthetic drug allergy tests were no positive findings.

After 2 months, the patient was readmitted for the cranioplasty. The medication for underlying diseases remained the same. However, azilsartan was discontinued 4 days before general anesthesia, and the anesthetic induction agent was switched from propofol to remimazolam to prevent hypotension during anesthesia in this case. In the operating room, the initial vital signs were as follows; BP 171/88 mm Hg, HR 58 bpm, oxygen saturation 97% at room air. An arterial cannulation was performed for invasive BP monitoring, and cardiogenic drugs including ephedrine, norepinephrine, and vasopressin before anesthesia induction. General anesthesia was induced with remimazolam 5 mg, rocuronium 60 mg. Anesthesia was maintained with desflurane and remifentanil. Approximately 15 minutes after endotracheal intubation, the BP decreased to 80/37 mm Hg with a HR of 49 bpm. As a result, continuous infusion of norepinephrine (0.05 mcg/kg/min) was started. Afterward, the BP maintained stable during the surgery, and norepinephrine infusion was discontinued before the end of anesthesia. The patient recovered from anesthesia without complications and was transferred to the post-anesthetic recovery room.

## 3. Discussion

RAAS is one of the 3 major systems, along with the sympathetic nervous system and the vasopressin system, responsible for maintaining hemodynamic stability. General anesthesia suppresses the sympathetic nervous system, during which RAAS plays a crucial role in maintaining hemodynamic stability. However, RAAS inhibitors interfere with sympathetic vasoconstrictor responses and enhance parasympathomimetic effects. As a result, they lead to vasodilation, decreased heart rate responses, and reduced responsiveness to exogenous catecholamines, which can cause severe refractory hypotension during general anesthesia-induced sympathetic nervous system suppression.^[10]^ There are guidelines regarding the use of RAAS inhibitors before surgery to prevent intraoperative hypotension. However, the issue of whether to discontinue or continue ARBs before surgery remains controversial.

The most recent guidelines from the European Society of Cardiology recommend that withholding ARBs on the day of surgery should be considered to prevent perioperative hypotension in patients without heart failure, while recommending continued use in patients with stable heart failure.^[5]^ The 2019 Perioperative quality initiative consensus statement also suggests withholding ARBs 24 hours before surgery unless clinically contraindicated and restarting the medication within 48 hours after surgery if possible.^[4]^ In 2014, the American College of Cardiology/American Heart Association recommended continuing angiotensin axis blockade before surgery.^[11]^ However, in 2017, the guidelines regarding the use of RAAS inhibitors before surgery were updated, and they suggested that discontinuing ARBs perioperatively may be considered in patients undergoing major surgery.^[1]^ These community recommendations are still limited due to lack of clear supporting evidence from research. Currently, randomized controlled trials such as the STOP-or-NOT trial (NCT03374449) and the POISE-3 trial (NCT03505723) are ongoing, and these studies may provide more definitive guidelines.^[12,13]^

The optimal duration of preoperative discontinuation of ARBs to prevent intraoperative hypotension is still unclear. Although the half-life of ARBs varies from 6 to 24 hours, and most ARBs have a duration of action of more than 24 hours,^[10]^ the general practice is uniformly to discontinue ARBs for 24 hours before surgery.^[1,3–5]^ However, several reports have shown refractory hypotension occurring during general anesthesia even after a 24-hour discontinuation of ARBs.^[6–9]^ Some reports suggest that prolonging the medication discontinuation period to 2 to 5 days can secure hemodynamic stability during anesthesia.^[7–9]^ This indicates the need for new criteria for the duration of ARBs discontinuation.

Hojo et al reported refractory hypotension associated with azilsartan in patients with renal impairment and stated that refractory hypotension could be avoided by discontinuing the medication for 48 hours before surgery.^[9]^ Azilsartan is the last ARB approved by the US FDA in 2011 and has superior ability in controlling 24-hour systolic blood pressure compared to other ARBs.^[14]^ The half-life of azilsartan is 13 hours. A 32 mg dose of azilsartan reduces the maximal pressor effect of angiotensin II by approximately 90% when reaches peak plasma concentration, and the pressor effect is reduced by approximately 60% after 24 hours of drug administration.^[15]^ In present case, the patient was taking 80 mg of azilsartan once daily and discontinued it for 48 hours before surgery. However, severe refractory hypotension occurred during anesthesia induction. For the second surgery, ARBs discontinuation period was extended to 96 hours, refractory hypotension did not occur, although there was post-induction hypotension that was controlled with continuous infusion of norepinephrine. ARBs-induced hypotension attenuates the effects of phenylephrine and ephedrine, requiring repeated administration and large doses of these pressors. In cases of refractory hypotension that do not respond to these pressors, vasopressin or terlipressin may be useful.^[10]^ Additionally, propofol during anesthesia induction has a potent vasodilatory effect and resets the baroreceptor reflex downward, increasing the risk of hypotension. Recent study has shown that remimazolam causes less post-induction hypotension in patients using angiotensin axis blockade compared to propofol.^[16]^ Furthermore, desflurane increases the activity of the sympathetic nervous system, making it suitable for maintenance anesthesia in patients of ARBs.^[10]^ In our case, the discontinuation period of ARBs was extended to 96 hours before the second surgery to prevent refractory hypotension and remimazolam was used for anesthesia induction and desflurane was used for maintenance anesthesia. Vasopressin was also prepared before anesthesia induction.

In this study, one limitation is the non-administration of vasopressin during refractory hypotension occurring in the first anesthesia. If refractory hypotension had been corrected by vasopressin, it could have provided a more definitive diagnosis of refractory hypotension during anesthesia caused by ARBs, and surgery postponement might not have been necessary.

## 4. Conclusions

Despite discontinuing azilsartan 48 hours before surgery, severe refractory hypotension occurred during anesthesia induction. However, extending the discontinuation period to 96 hours before the second surgery prevented refractory hypotension during general anesthesia. Well-designed studies are needed to determine the appropriate preoperative discontinuation period for ARBs.

## Author contributions

**Conceptualization:** Yu Yil Kim.

**Investigation:** Ji Hye Lee, Hyun Joo Heo.

**Resources:** Gwanbeom Kim, Changhwan Oh.

**Supervision:** Ji Hye Lee, Yu Yil Kim.

**Writing – original draft:** Ji Hye Lee, Yu Yil Kim.

**Writing – review & editing:** Ji Hye Lee, Yu Yil Kim, Hyun Joo Heo.
